# Supplementary material for: Adaptive Evolution of Animal Proteins over Development: Support for the Darwin Selection Opportunity Hypothesis of Evo-Devo
Source: Mol Biol Evol. 2018 Sep 1;35(12):2862–72. doi: 10.1093/molbev/msy175 (PMC6278863; doi:10.1093/molbev/msy175)
Supplement: Supplementary Data [file msy175_supp.zip › Supp.docx]

**Figure S1: Expression profiles of different modules across development.**

The bold black line represents median expression of modular genes, the two gray lines represent 25th and 75th quantiles of expression of modular genes respectively. The blue, red and green named stages represent early, middle and late modules respectively.

**Figure S2: Proportion of genes with strong evidence of positive selection in each module.**

The number of genes in each module is indicated below each box. The *p*-value from chi-square goodness of fit test is reported in the top-left corner of each graph.

**Figure S3: Proportion of genes with weak evidence of positive selection in each module.**

Legend as in Figure S2.

**Figure S4: Spearman’s correlation between gene properties and ΔlnL.**

Spearman’s correlation coefficient (rho) and adjusted *p*-value are indicated in the top-right corner of each graph. Loess regression lines are plotted in red.

**Figure S5: Variation of gene length in different modules.**

Legend as in Figure 1.

**Figure S6: Transcriptome index of gene length across development.**

Legend as in Figure 2.

**Figure S7: Transcriptome index of ΔlnL (TLI) for non-immune genes.**

Legend as in Figure 2.

**Figure S8: Transcriptome index of ΔlnL (TLI) for non-testis genes in *M. musculus* and *D. melanogaster*.**

Legend as in Figure 2.

**Figure S9: Scatter plot of genes based on principal component analysis**

Each dot represents one gene, grey dots represent genes not assigned to any modules, blue dots represent genes in early embryo module, red dots represent genes in middle embryo module, blue dots represent genes in late embryo module, pink dots represent genes in larva module, and purple dots represent genes in pupae/adult module. Arrow indicates the gene expression order (from early to late).

**Figure S10: Heat map of gene expression across development**

Genes arranged by the order of expression (from early to late). Generally, the earlier genes have higher expression in earlier stages. Color bar represents the standardized expression value.

**Figure S11: idealized expression profile for each module.**

**Figure S12: Correction of gene length for polygenic selection.**

The red lines indicate the boundaries of bins which contains genes with similar length.
