# Supplementary material for: Adaptive Evolution of Animal Proteins over Development: Support for the Darwin Selection Opportunity Hypothesis of Evo-Devo
Source: Mol Biol Evol. 2018 Sep 1;35(12):2862–72. doi: 10.1093/molbev/msy175 (PMC6278863; doi:10.1093/molbev/msy175)
Supplement: Supplementary Data [file msy175_supp.zip › SupplementaryFiguresLiu.pdf]

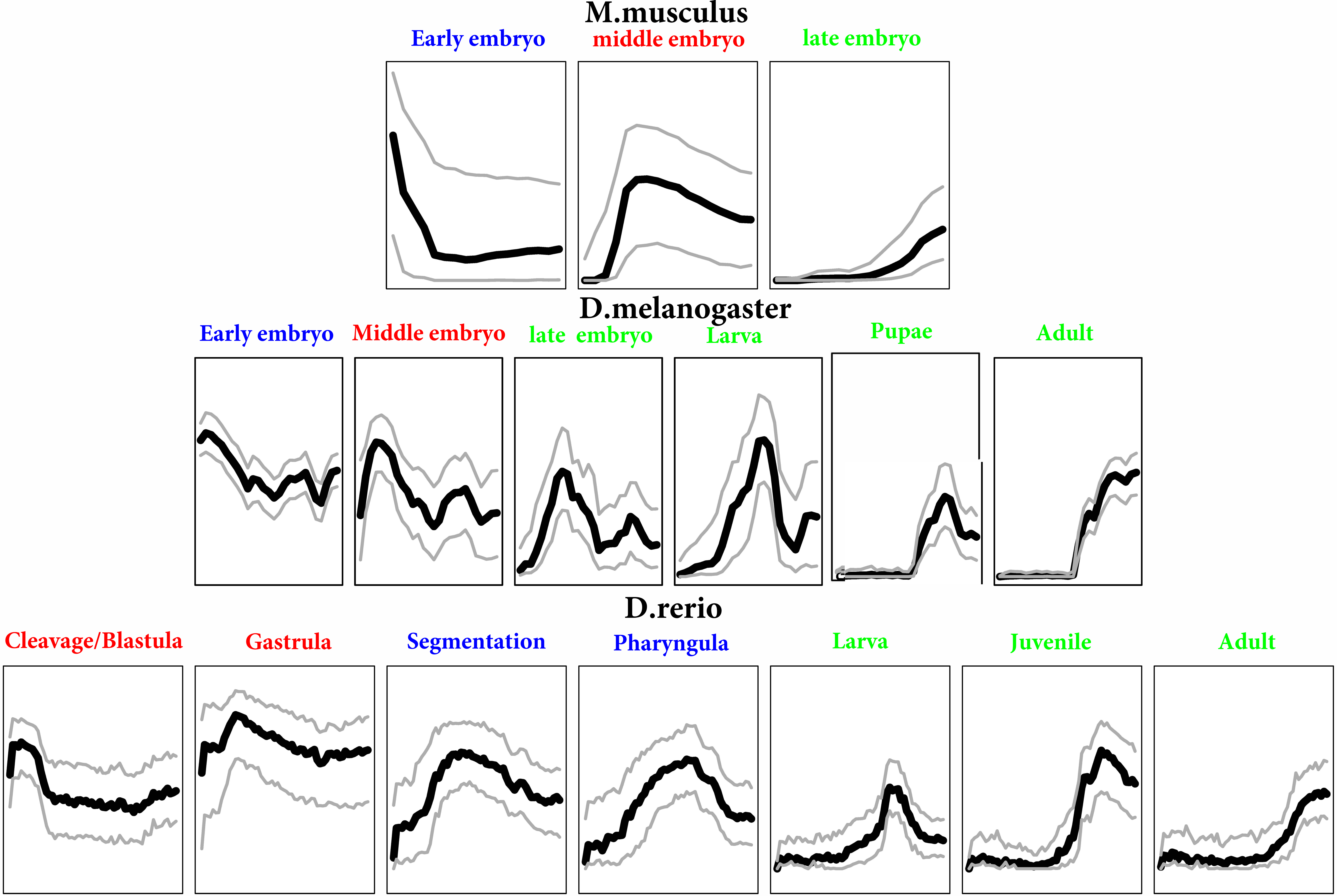
M.musculus
Early embryo
middle embryo
late embryo
D.melanogaster
Early embryo
Middle embryo
late embryo
Larva
Pupae
Adult
D.rerio
Cleavage/Blastula
Gastrula
Segmentation
Pharyngula
Larva
Juvenile
Adult

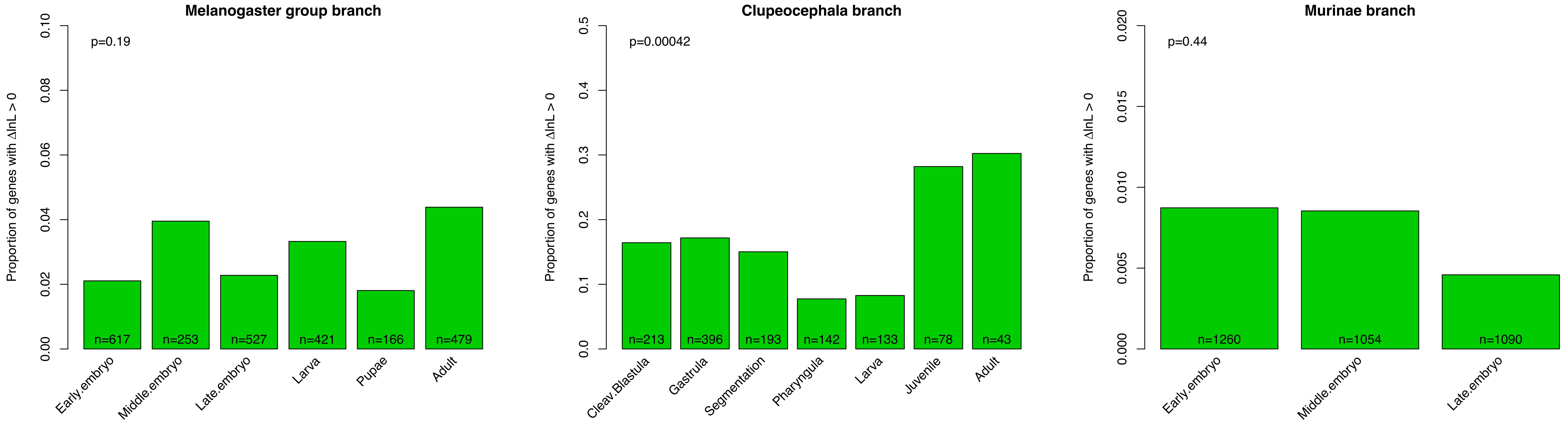

Melanogaster group branch
p=0.19
Proportion of genes with ΔlnL > 0
0.00
0.02
0.04
0.06
0.08
0.10
n=617
n=253
n=527
n=421
n=166
n=479
Early.embryo
Middle.embryo
Late.embryo
Larva
Pupae
Adult
Clupeocephala branch
p=0.00042
Proportion of genes with ΔlnL > 0
0.0
0.1
0.2
0.3
0.4
0.5
n=213
n=396
n=193
n=142
n=133
n=78
n=43
Cleav.Blastula
Gastrula
Segmentation
Pharyngula
Larva
Juvenile
Adult
Murinae branch
p=0.44
Proportion of genes with ΔlnL > 0
0.000
0.005
0.010
0.015
0.020
n=1260
n=1054
n=1090
Early.embryo
Middle.embryo
Late.embryo

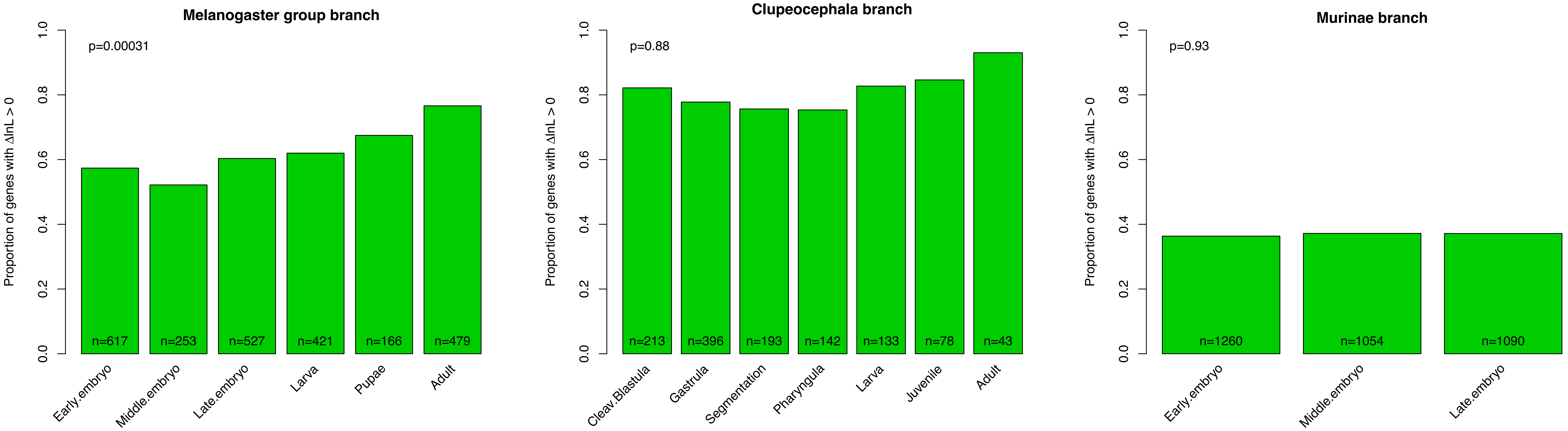

Melanogaster group branch
p=0.00031
Proportion of genes with ΔlnL > 0
1.0
0.8
0.6
0.4
0.2
0.0
n=617
n=253
n=527
n=421
n=166
n=479
Early.embryo
Middle.embryo
Late.embryo
Larva
Pupae
Adult
Clupeocephala branch
p=0.88
n=213
n=396
n=193
n=142
n=133
n=78
n=43
Cleav.Blastula
Gastrula
Segmentation
Pharyngula
Larva
Juvenile
Adult
Murinae branch
p=0.93
n=1260
n=1054
n=1090
Early.embryo
Middle.embryo
Late.embryo

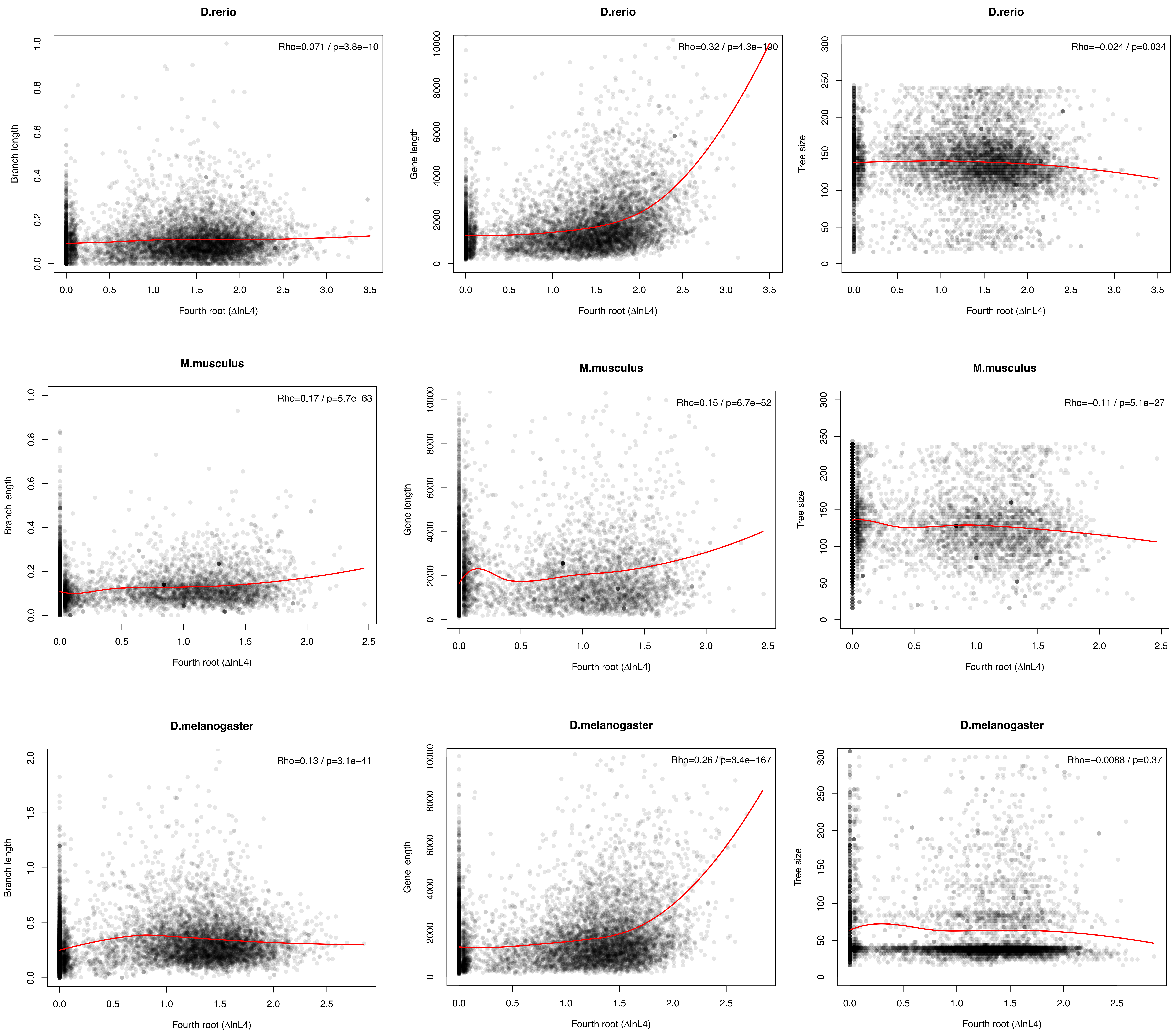
D.rerio
Rho=0.071 / p=3.8e−10
Branch length
Fourth root (ΔlnL4)
D.rerio
Rho=0.32 / p=4.3e−190
Gene length
Fourth root (ΔlnL4)
D.rerio
Rho=−0.024 / p=0.034
Tree size
Fourth root (ΔlnL4)
M.musculus
Rho=0.17 / p=5.7e−63
Branch length
Fourth root (ΔlnL4)
M.musculus
Rho=0.15 / p=6.7e−52
Gene length
Fourth root (ΔlnL4)
M.musculus
Rho=−0.11 / p=5.1e−27
Tree size
Fourth root (ΔlnL4)
D.melanogaster
Rho=0.13 / p=3.1e−41
Branch length
Fourth root (ΔlnL4)
D.melanogaster
Rho=0.26 / p=3.4e−167
Gene length
Fourth root (ΔlnL4)
D.melanogaster
Rho=−0.0088 / p=0.37
Tree size
Fourth root (ΔlnL4)

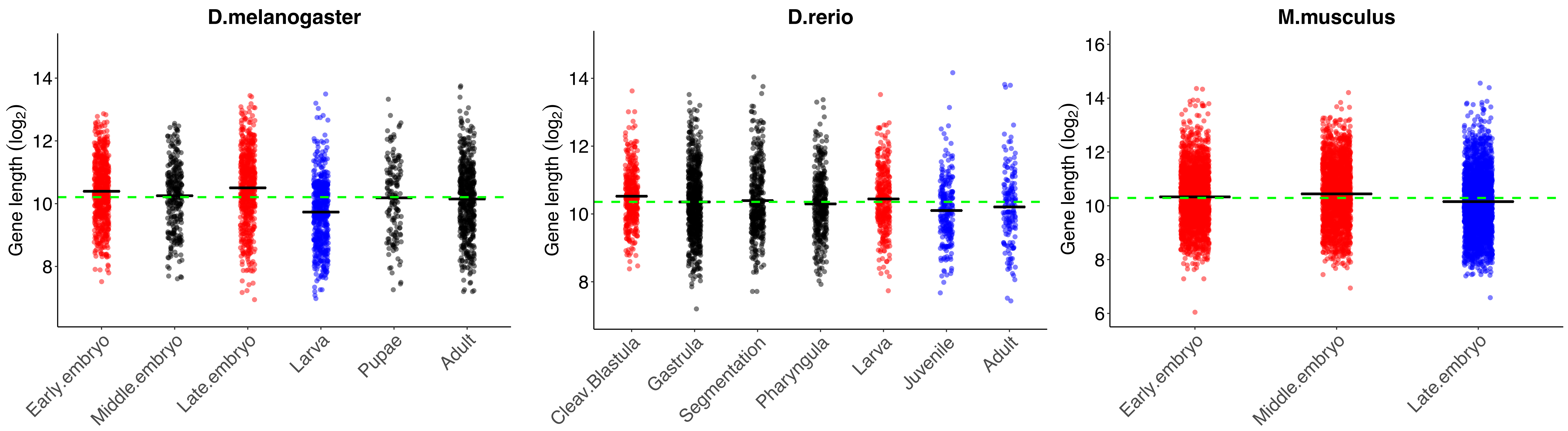
D.melanogaster
Gene length (log2)
14
12
10
8
Early.embryo
Middle.embryo
Late.embryo
Larva
Pupae
Adult
D.rerio
Gene length (log2)
14
12
10
8
Cleav.Blastula
Gastrula
Segmentation
Pharyngula
Larva
Juvenile
Adult
M.musculus
Gene length (log2)
16
14
12
10
8
6
Early.embryo
Middle.embryo
Late.embryo

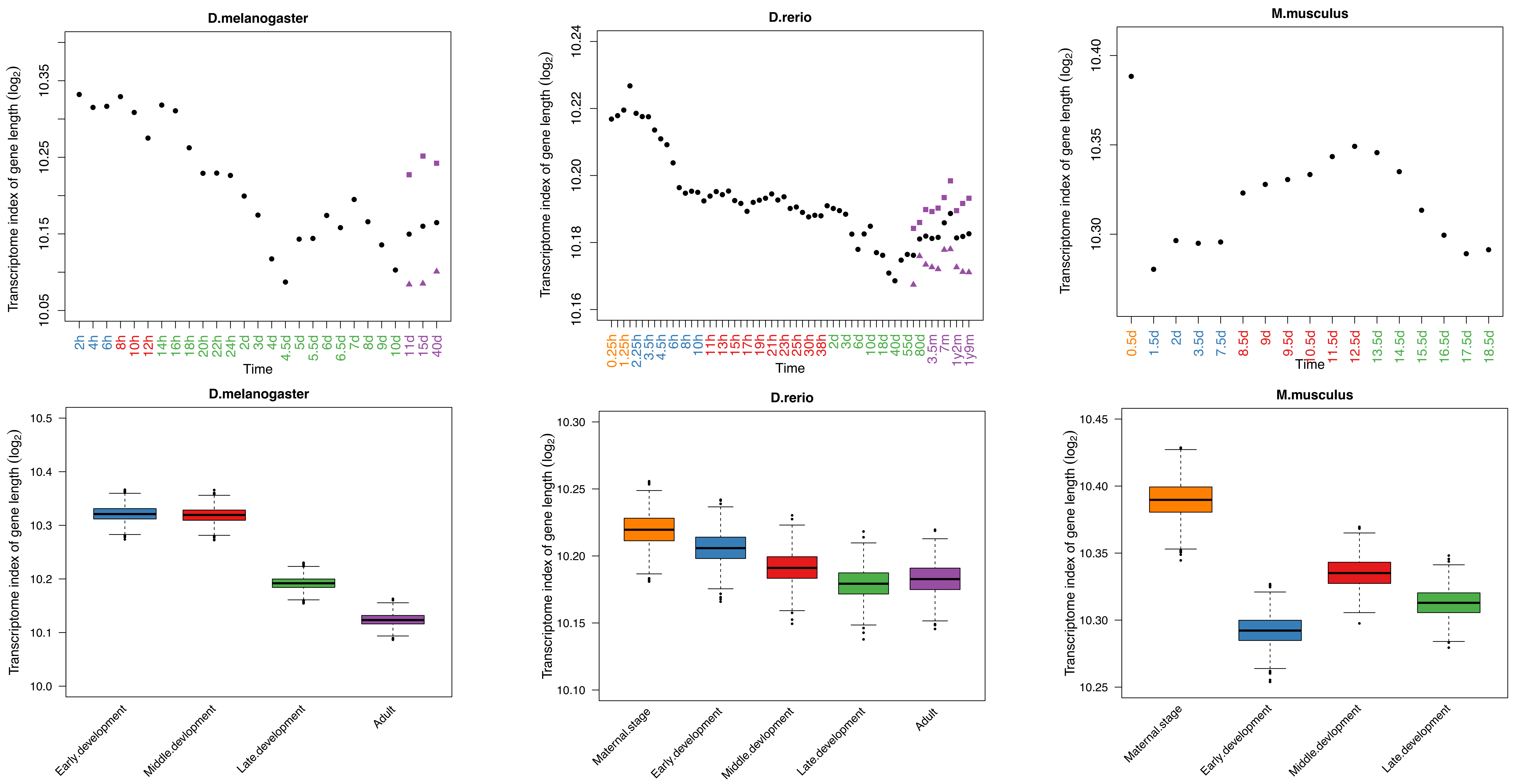

D.melanogaster
D.rerio
M.musculus
Transcriptome index of gene length (log2)
Time
2h 4h 6h 8h 10h 12h 14h 16h 18h 20h 22h 24h 2d 3d 4d 4.5d 5d 5.5d 6d 6.5d 7d 8d 9d 10d 11d 15d 40d
0.25h 1.25h 2.25h 3.5h 4.5h 6h 8h 10h 11h 13h 15h 17h 19h 21h 23h 25h 30h 38h 2d 3d 6d 10d 18d 40d 55d 80d 3.5m 7m 1y2m 1y9m
0.5d 1.5d 2d 3.5d 7.5d 8.5d 9d 9.5d 10.5d 11.5d 12.5d 13.5d 14.5d 15.5d 16.5d 17.5d 18.5d
Early.development
Middle.devlopment
Late.development
Adult
Maternal.stage

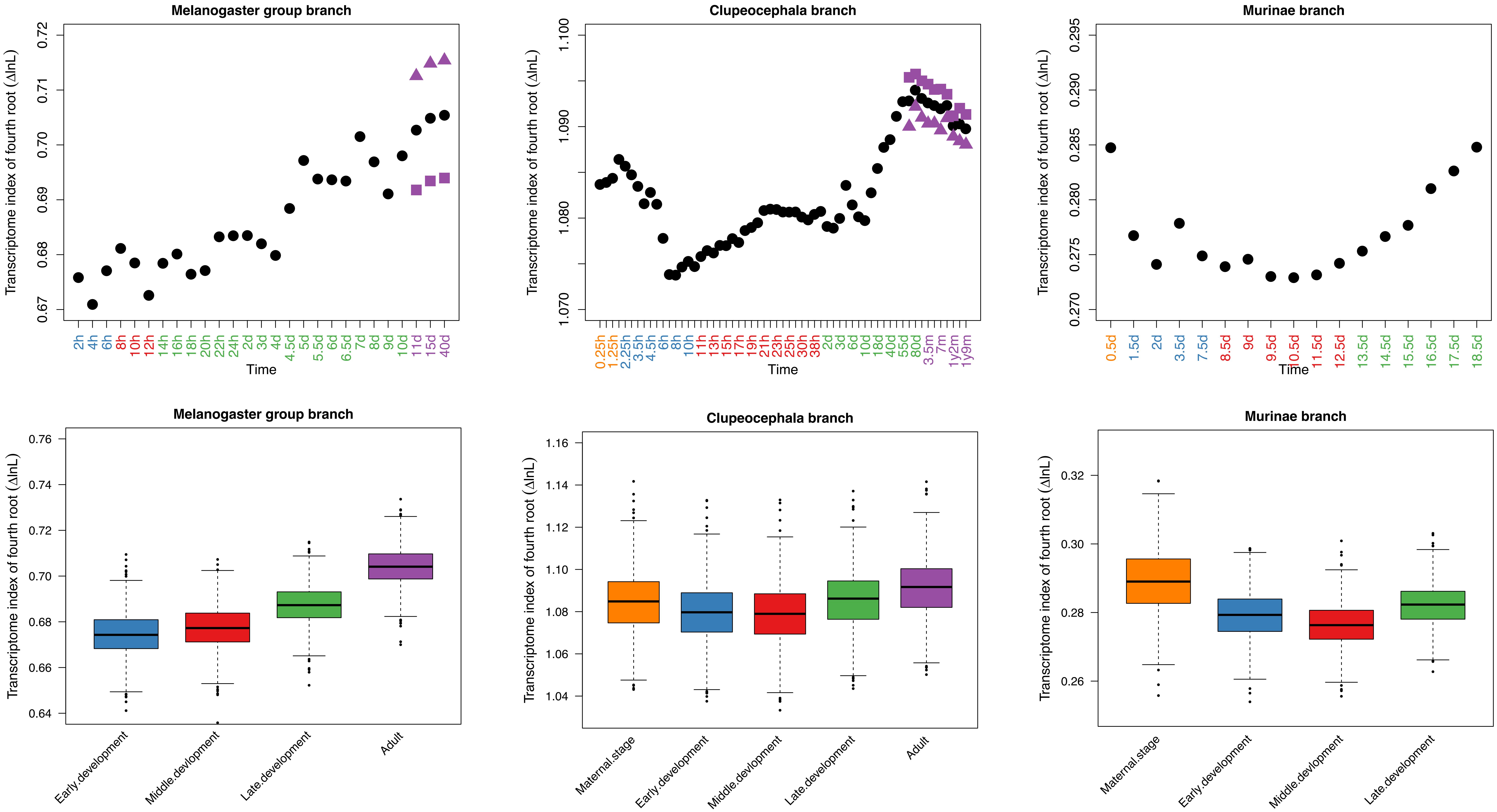

Melanogaster group branch
Transcriptome index of fourth root (ΔlnL)
0.67
0.68
0.69
0.70
0.71
0.72
2h 4h 6h 8h 10h 12h 14h 16h 18h 20h 22h 24h 2d 3d 4d 4.5d 5d 5.5d 6d 6.5d 7d 8d 9d 10d 11d 15d 40d
Time
Clupeocephala branch
Transcriptome index of fourth root (ΔlnL)
1.070
1.080
1.090
1.100
0.25h 1.25h 2.25h 3.5h 4.5h 6h 8h 10h 11h 13h 15h 17h 19h 21h 23h 25h 30h 38h 2d 3d 6d 10d 18d 40d 55d 80d 3.5m 7m 1y2m 1y9m
Time
Murinae branch
Transcriptome index of fourth root (ΔlnL)
0.270
0.275
0.280
0.285
0.290
0.295
0.5d 1.5d 2d 3.5d 7.5d 8.5d 9d 9.5d 10.5d 11.5d 12.5d 13.5d 14.5d 15.5d 16.5d 17.5d 18.5d
Time
Melanogaster group branch
Transcriptome index of fourth root (ΔlnL)
0.64
0.66
0.68
0.70
0.72
0.74
0.76
Early.development
Middle.devlopment
Late.development
Adult
Clupeocephala branch
Transcriptome index of fourth root (ΔlnL)
1.04
1.06
1.08
1.10
1.12
1.14
1.16
Maternal.stage
Early.development
Middle.devlopment
Late.development
Adult
Murinae branch
Transcriptome index of fourth root (ΔlnL)
0.26
0.28
0.30
0.32
Maternal.stage
Early.development
Middle.devlopment
Late.development

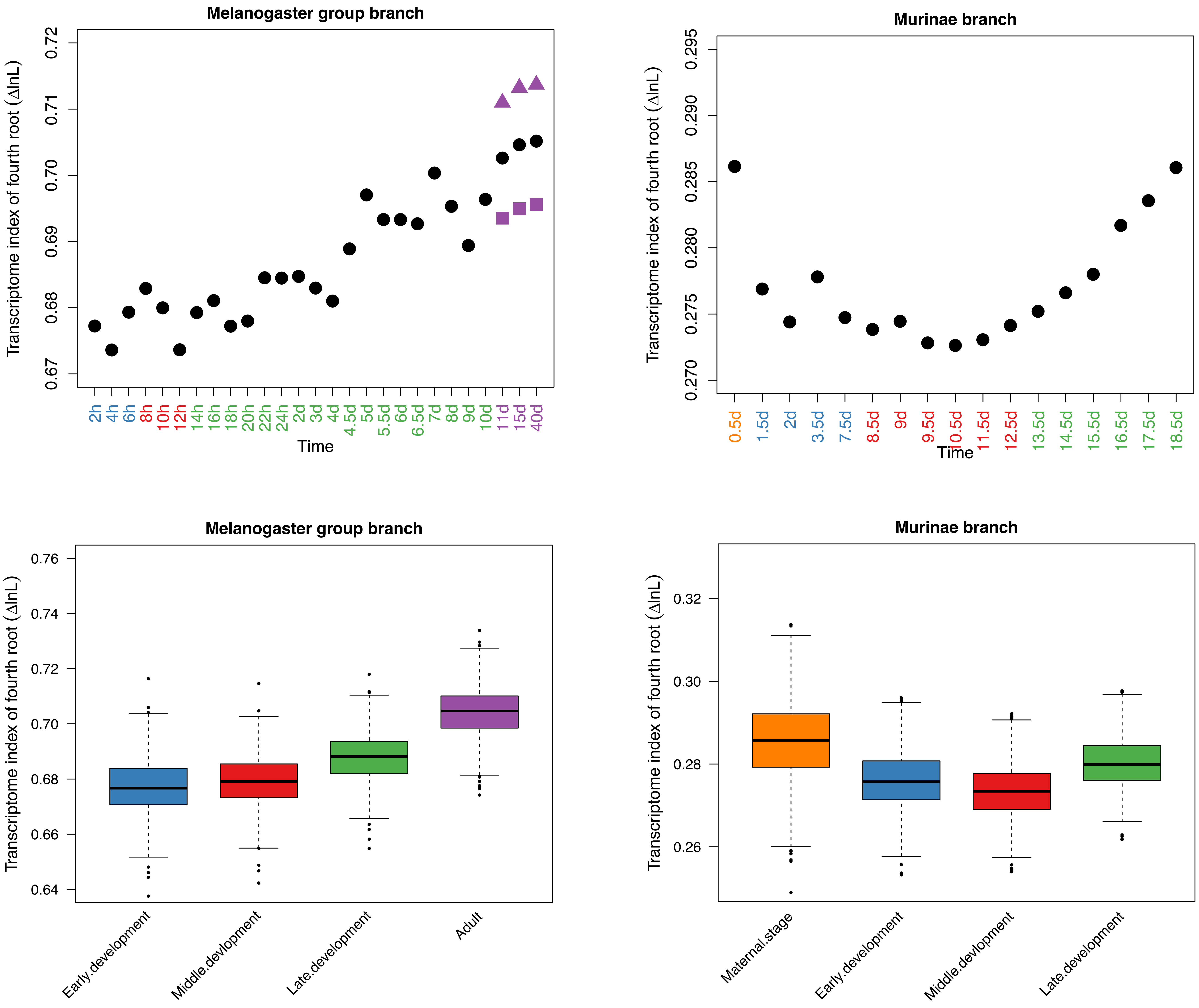

Melanogaster group branch
Transcriptome index of fourth root (ΔlnL)
Time
2h 4h 6h 8h 10h 12h 14h 16h 18h 20h 22h 24h 2d 3d 4d 4.5d 5d 5.5d 6d 6.5d 7d 8d 9d 10d 11d 15d 40d
Murinae branch
0.5d 1.5d 2d 3.5d 7.5d 8.5d 9d 9.5d 10.5d 11.5d 12.5d 13.5d 14.5d 15.5d 16.5d 17.5d 18.5d
Early.development
Middle.devlopment
Late.development
Adult
Maternal.stage

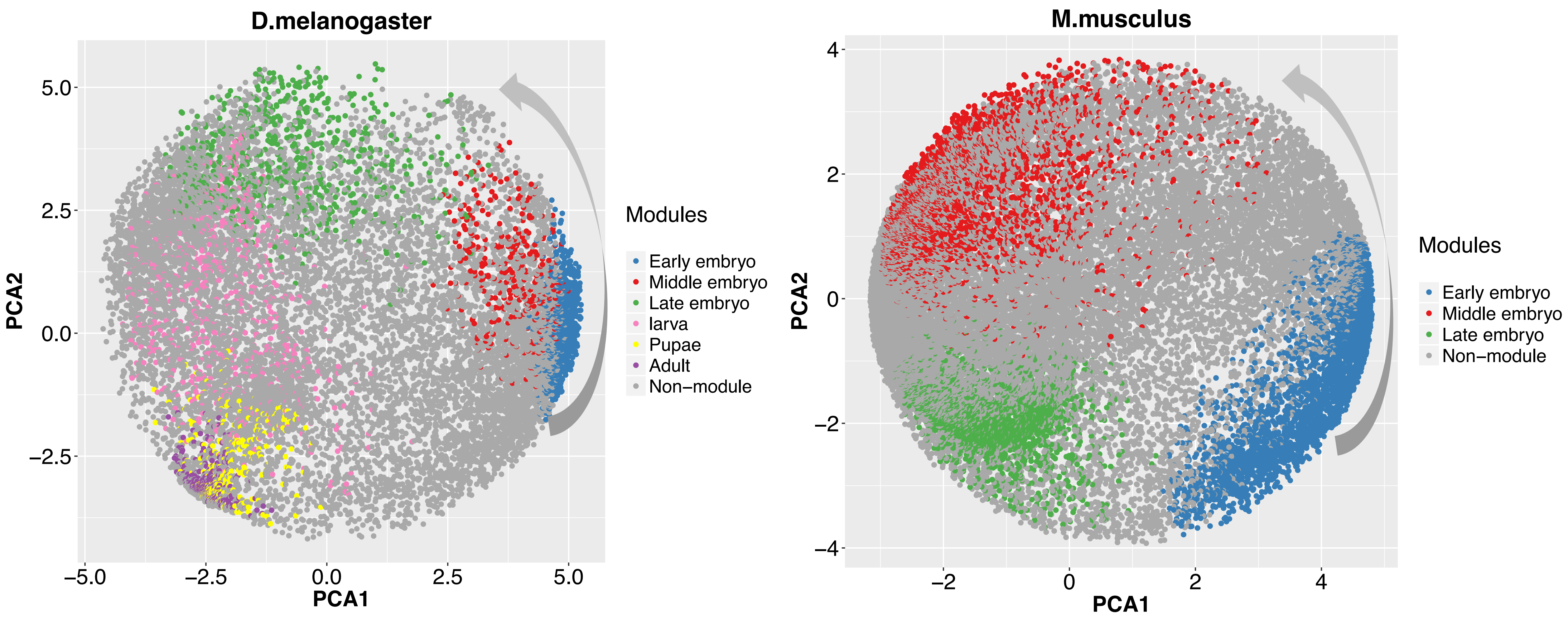
D.melanogaster
5.0
2.5
0.0
−2.5
−5.0
−2.5
0.0
2.5
5.0
PCA1
PCA2
Modules
Early embryo
Middle embryo
Late embryo
larva
Pupae
Adult
Non−module
M.musculus
4
2
0
−2
−4
−2
0
2
4
PCA1
PCA2
Modules
Early embryo
Middle embryo
Late embryo
Non−module

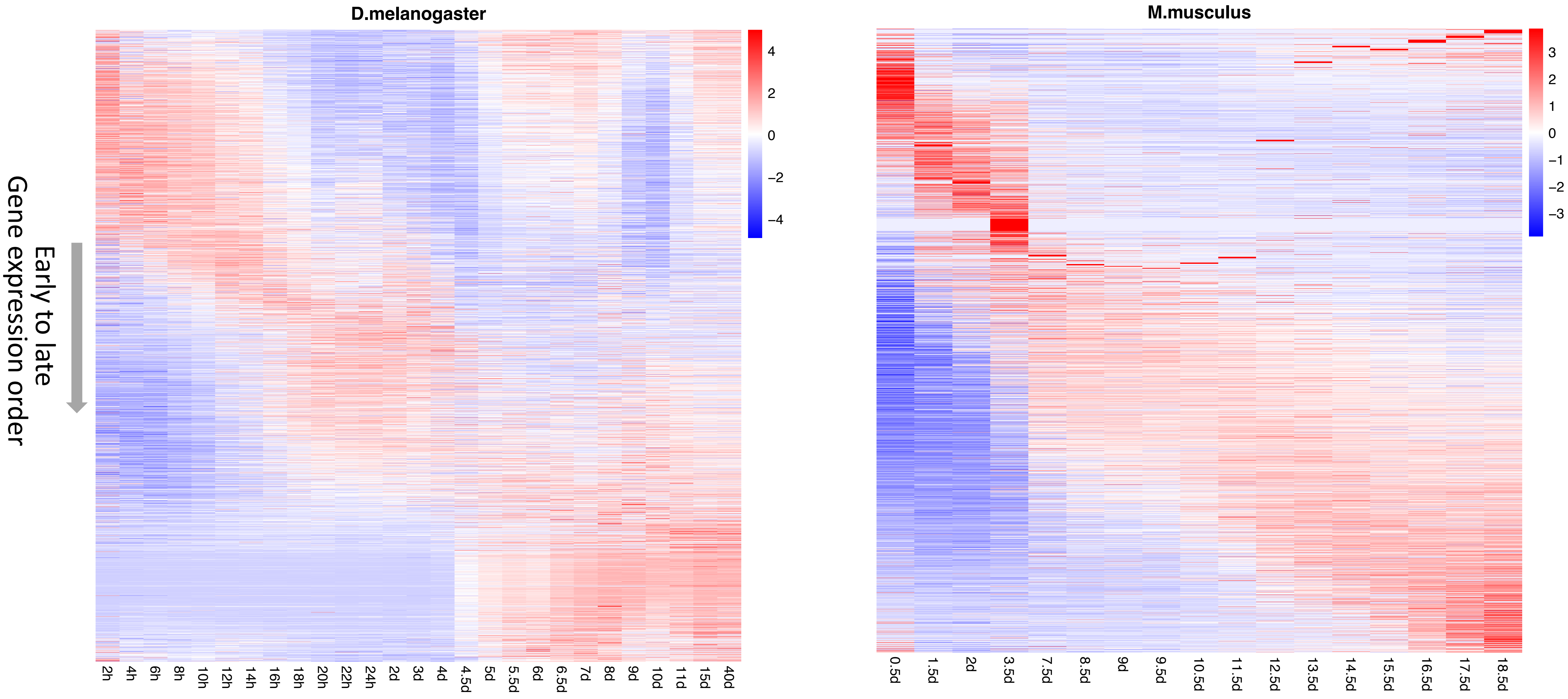

D.melanogaster
M.musculus
Early to late
Gene expression order
4
2
0
−2
−4
3
2
1
0
−1
−2
−3
2h
4h
6h
8h
10h
12h
14h
16h
18h
20h
22h
24h
2d
3d
4d
4.5d
5d
5.5d
6d
6.5d
7d
8d
9d
10d
11d
15d
40d
0.5d
1.5d
2d
3.5d
7.5d
8.5d
9d
9.5d
10.5d
11.5d
12.5d
13.5d
14.5d
15.5d
16.5d
17.5d
18.5d

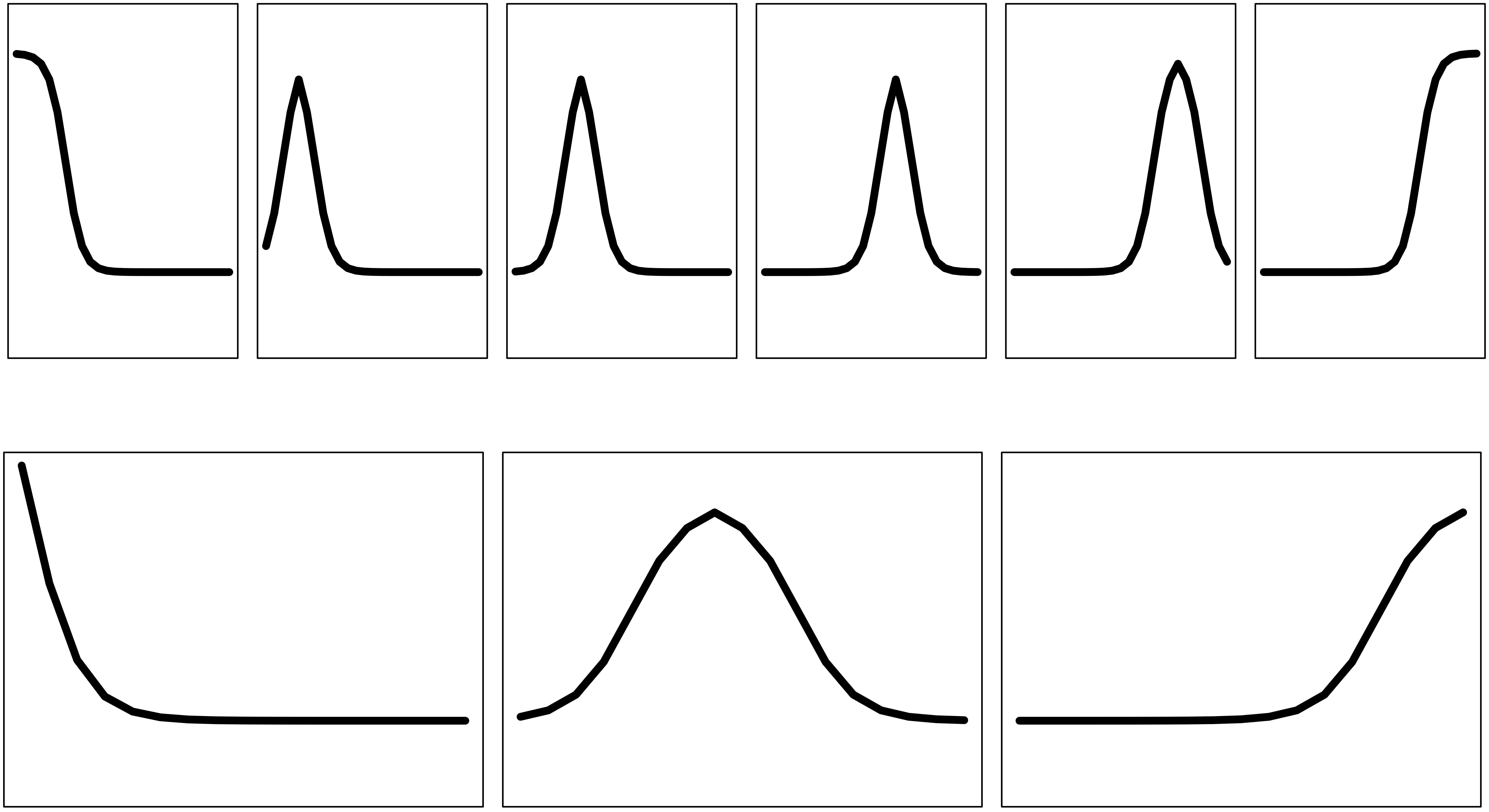

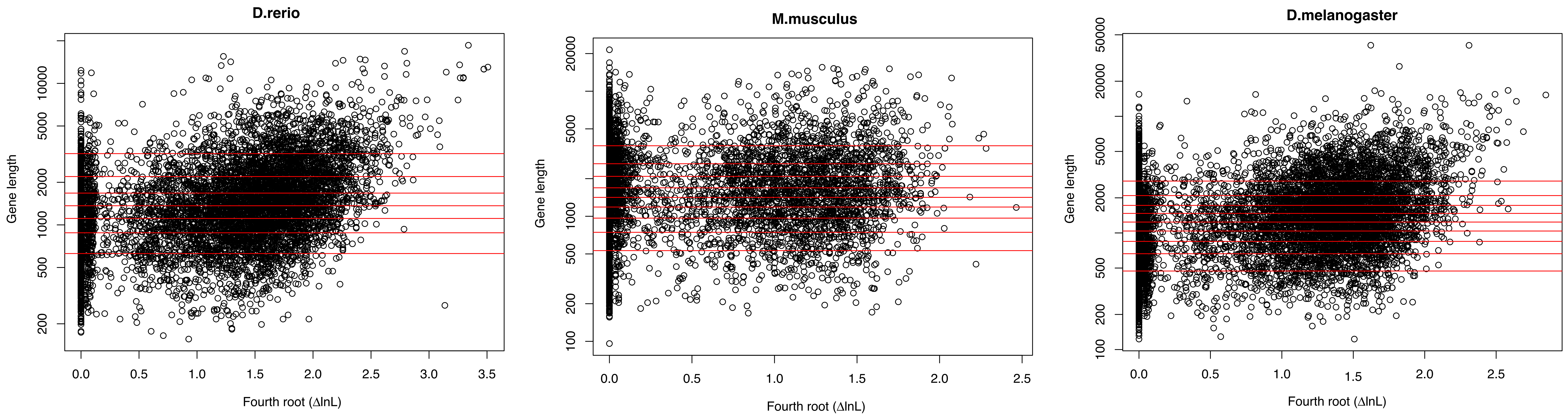

D.rerio
M.musculus
D.melanogaster
Gene length
Fourth root (ΔlnL)
200
500
1000
2000
5000
10000
20000
50000
100
0.0
0.5
1.0
1.5
2.0
2.5
3.0
3.5
